# Effects of a Single Venous Dose of  Zinc on Thyroid Status in Healthy Individuals and Patients With Graves' Disease

**DOI:** 10.1155/MBD.2000.151

**Published:** 2000

**Authors:** Lubna Farooqi, Gláucia M. F. S. Mazeto, Tadao Shuhama, José Brandão-Neto

**Affiliations:** Unidade de Endocrinologia e Metabologia Faculdade de Ciências da Saúde Universidade de Brasilia DF 70 919-970 Brazil

## Abstract

Zinc metabolism may regulate thyroid function acting at TRH (thyrotropin-releasing hormone) synthesis, peripheral deiodination of T4 (tetraiodothyronine), and binding of thyroid hormones to nuclear receptors. The aim of this study was to investigate the effect of acute zinc administration on TSH (thyroid-stimulating hormone), FT3 (free triiodothyronine), and FT4 (free tetraiodothyronine) in 10 healthy individuals and 12 hyperthyroid patients with Graves' disease. All these individuals were studied following 25 mg Zn^++^ administered intravenously, at 7:00 a.m. after 12 h fast. Blood samples collected at 0, 3, 30, 60, 90, and 120 min after zinc administration showed no significant alteration in the plasma levels of TSH, FT3, and FT4 in hyperthyroid patients. There were no changes in the plasma levels of FT3 and FT4 in the control subjects, but TSH levels were acutely depressed by zinc administration. This study suggests that zinc given acutely and in pharmacological doses does not affect thyroid function in hyperthyroid subjects, but affect plasma TSH levels in healthy individuals.


### EFFECTS OF A SINGLE VENOUS DOSE OF ZINC ON THYROID STATUS IN HEALTHY INDIVIDUALS AND PATIENTS WITH GRAVES' DISEASE

Lubna Farooqi, Gláucia M.F.S. Mazeto, Tadao Shuhama and José Brandão-Neto*

Unidade de Endocrinologia e Metabologia, Faculdade de Ciências da Saúde, Universidade de Brasília, Brasília - DF, CEP: 70 919-970, CP: 4591 <jbn@brnet.com.br>

### Abstract

Zinc metabolism may regulate thyroid function acting at TRH (thyrotropin-releasing hormone) synthesis, peripheral deiodination of T4 (tetraiodothyronine), and binding of thyroid hormones to nuclear receptors. The aim of this study was to investigate the effect of acute zinc administration on TSH (thyroid-stimulating hormone), FT3 (free triiodothyronine), and FT4 (free tetraiodothyronine) in 10 healthy individuals and 12 hyperthyroid patients with Graves' disease. All these individuals were studied following 25 mg $Zn^{++}$ administered intravenously, at 7:00 a.m. after 12 h fast. Blood samples collected at 0, 3, 30, 60, 90, and 120 min after zinc administration showed no significant alteration in the plasma levels of TSH, FT3, and FT4 in hyperthyroid patients. There were no changes in the plasma levels of FT3 and FT4 in the control subjects, but TSH levels were acutely depressed by zinc administration. This study suggests that zinc given acutely and in pharmacological doses does not affect thyroid function in hyperthyroid subjects, but affect plasma TSH levels in healthy individuals.

### Introduction

Graves' disease is the most common form of thyrotoxicosis. It occurs at any age and is more frequent in females. It is currently viewed as an autoimmune disease of unknown cause. The pathogenesis is related to T lymphocytes that become sensitized to antigens within the thyroid gland and stimulate B lymphocytes to synthesize antibodies to these antigens. TSHRAb (antibody against the thyroid-stimulating hormone receptor) is one such antibody that acts in the thyroid cell membrane and has the capacity to stimulate the thyroid cell to increase growth and function [1]. Thus, Graves' disease consists of one or more of the following features: thyrotoxicosis, goiter, exophthalmos, and pretibial myxedema [1].

Zinc is a metal that affects thyroid hormone function at several levels. For example, zinc deficiency inhibits TRH synthesis [2,3] and depresses plasma TSH, T4, T3 (triiodothyronine) [2,3]. It is necessary for extrathyroidal T4 to T3 conversion [2,4], and it plays a role in T3 binding to nuclear receptor as well as the binding of the receptor to DNA [5].

On the other hand, the interaction between thyroid hormone and zinc metabolism has also been reported, but the data on these topics are still limited [6,7,8]. Thus, hypo- or hyperthyroidism can affect the absorption, transport, secretion and excretion of zinc [9,10]. Moreover, chronic zinc administration has been found to affect the circulating levels of TSH, T3, T4, and rT3 (reverse triiodothyronine) [11]. There appears to be a close relationship between zinc and thyroid hormones, but the interactive mechanism is unknown. A few studies have been reported on thyroid metabolism in patients with thyroid diseases and associated zinc deficiency [8], but no previous reports are available on thyroid status following acute zinc administration in patients with Graves' disease.

In this study, we determined whether a single venous dose of zinc changes the plasma TSH, FT3, and FT4 in healthy individuals and in hyperthyroid patients.

### Material and methods

### *Subjects*

The study group was comprised of 10 healthy individuals (5 of each sex, aged 24.10 ± 1.96) and 12 hyperthyroid patients with Graves' disease (1 male and 11 females, aged 27.25 ± 7.31). Healthy individuals (medical students or staff members) were free from endocrine disease, and none used any type of medication. Clinical evidence and biochemical determination of TSH, FT3, and FT4 confirmed the thyroid status of hyperthyroidism. Hyperthyroid patients received no antithyroid drugs or beta-adrenergic blocker before this study, and they did not have other endocrine complications. Duration of hyperthyroidism was recent (no longer than 3 mo). None of the subjects were on a special diet, and were taking any form of vitamin or mineral supplements. The protocol, which was approved by the Medical Ethics Committee, was explained to the patients and written consent was obtained from all participants.

*Experimental design*
Group 1 (control): Ten healthy individuals.
Group 2 (experimental): Twelve hyperthyroid patients.

*Venous zinc tolerance test*
This test was started at 7:00 a.m. after 12 h fast, with the subjects maintaining dorsal decubitus throughout the test. Catheters for infusion were implanted into the antecubital veins of both forearms and maintained with physiological saline. Basal blood samples were collected at -30 and 0 min for zinc, TSH, FT3, and FT4. Intravenous administration of 25 mg Zn++ (2 mL zinc sulfate) over a period of 1 min was started at time 0 min (8:00 h). This dose was used because although it is in the supraphysiological range, it is devoid of any toxic effects [12]. Blood samples were then immediately collected from another vein in the contralateral arm at 3, 30, 60, 90, and 120 min.

*Sample collection and analysis*
Venipuncture was performed using plastic syringes without a tourniquet. Materials used for zinc collection, separation, and storage are made of propylene plastic and metal free. Serum and plasma samples were frozen and stored at -20 °C until the time of measurement. We determined zinc concentration in serum, not in plasma, to avoid the use of anticoagulants that are commonly a source of zinc contamination. It was measured by atomic absorption spectrophotometer (Shimadzu, AA680G, Japan) according to the instructions of manufacturers. Hemolyzed samples were discarded. The intraassay error was 2.4% and sensitivity was 0.02 µg/mL. Plasma TSH, FT3, and FT4 were measured by radioimmunoassay (Diagnostic Products Corporation, USA), with an intraassay error of 3.8, 4.7, and 1.4%, and a sensitivity of 0.05 µIU/mL, 0.02 pg/mL, and 0.1 pg/mL, respectively.

*Statistical analysis*
Student's *t* test, linear regression test, and correlation analysis were used for statistical assessments. All comparisons are considered to be statistically significant at the 5% significant level ($p<0.05$).

### Results

*Zinc dose, body surface area (BSA) and body mass index (BMI)*
We corrected the dose 25 mg $Zn^{++}$ by the BSA and found no significant difference between the means of Groups 1 and 2, $p>0.05$ (Table I). Both the means BSA and BMI are not significantly different between Groups 1 and 2, $p>0.05$ (Table I).

Table I: Values of zinc dose (25 mg elemental zinc corrected to BSA of each control and hyperthyroid patient), AUC (obtained from Table II and determined by the trapezoid rule), BSA (obtained from 1.73/m²), and BMI (obtained from weight (kg)/height² (m²)). These parameters were obtained from 10 healthy individuals and 12 hyperthyroid patients with Graves' disease.

| | GROUP 1 (Control) | GROUP 2 (Experimental) |
|---|---|---|
| ZINC DOSE (mg $Zn^{++}/m^2$) | 22.86 ± 2.58 | 21.70 ± 2.12 |
| $^a p$ value | | $p>0.05$ |
| AUC ($mL.min^{-1}$) | 527.23 ± 70.12 | 471.59 ± 70.34 |
| $^b p$ value | | $p>0.05$ |
| BSA ($m^2$) | 1.11 ± 0.12 | 1.16 ± 0.12 |
| $^c p$ value | | $p>0.05$ |
| BMI ($kg/m^2$) | 19.41 ± 2.91 | 18.89 ± 3.70 |
| $^d p$ value | | $p>0.05$ |

The values are represented as mean ± SD for 10 healthy individuals in Group 1 and 12 hyperthyroid patients in Group 2.

a,b,c, and d Indicate no significant difference (p>0.05) between Group 1 and Group2 by unpaired *t* test.

*Serum zinc ($SZn^{++}$) concentration*
Basal serum zinc levels were in the normal range (0.7-1.2 µg/mL) and not different in controls (1.04 ± 0.22 µg/mL) and hyperthyroid patients (0.96 ± 0.19 µg/mL), $p>0.05$ (Table II). During the venous zinc tolerance test, $SZn^{++}$ levels showed the same profile in the two groups (Figure 1A and Table II), and there are no significant difference, by unpaired *t* test, between the area under the curve (AUC) from Group 1 and Group 2, $p>0.05$ (Table I).

Table II: Values of Zinc, TSH, FT3, and FT4 during a single venous dose of 25 mg of elemental zinc in 10 control individuals and 12 hyperthyroid patients.

| | TIME (Min) | GROUP 1 (Control) | | GROUP 2 (Experimental) |
|---|---|---|---|---|
| ZINC (μg/mL) | 0 | 1.04 ± 0.22 | | 0.96 ± 0.19 |
| | 3 | 9.57 ± 2.28 | | 8.03 ± 1.10 |
| | 30 | 5.84 ± 0.80 | | 5.52 ± 0.97 |
| | 60 | 4.99 ± 0.67 | | 4.33 ± 0.77 |
| | 90 | 4.31 ± 0.65 | | 3.77 ± 0.59 |
| | 120 | 3.82 ± 0.57 | | 3.23 ± 0.69 |
| [a]$p$ value | | | *$p$>0.05 | |
| TSH (μIU/mL) | 0 | 2.11 ± 1.06 | | 0.07 ± 0.04 |
| | 3 | 2.03 ± 0.87 | | 0.10 ± 0.19 |
| | 30 | 1.69 ± 0.74 | | 0.06 ± 0.02 |
| | 60 | 1.58 ± 0.63 | | 0.11 ± 0.10 |
| | 90 | 1.52 ± 0.65 | | 0.14 ± 0.18 |
| | 120 | 1.37 ± 0.62 | | 0.06 ± 0.03 |
| [b]$p$ value | | | **$p$<0.0001 | |
| FT3 (pg/mL) | 0 | 2.46 ± 0.80 | | 28.85 ± 9.52 |
| | 3 | 2.53 ± 0.71 | | 29.34 ± 9.19 |
| | 30 | 2.30 ± 0.51 | | 31.46 ± 10.63 |
| | 60 | 2.31 ± 0.32 | | 29.72 ± 9.81 |
| | 90 | 2.42 ± 0.32 | | 30.00 ± 10.59 |
| | 120 | 2.45 ± 0.64 | | 29.96 ± 12.58 |
| [c]$p$ value | | | **$p$<0.0001 | |
| FT4 (pg/mL) | 0 | 12.39 ± 3.09 | | 98.67 ± 36.10 |
| | 3 | 12.01 ± 2.10 | | 96.46 ± 31.06 |
| | 30 | 12.25 ± 2.50 | | 98.62 ± 27.69 |
| | 60 | 12.50 ± 2.49 | | 103.17 ± 9.76 |
| | 90 | 13.91 ± 4.15 | | 102.34 ± 7.94 |
| | 120 | 12.85 ± 3.18 | | 100.59 ± 5.60 |
| [d]$p$ value | | | **$p$<0.0001 | |

The values are represented as mean ± SD.

[a] Indicates no significant difference ($p$>0.05) between Group 1 and Group 2 by unpaired $t$ test.

[b, c, and d] Indicate a significant difference ($p$<0.0001) between Group 1 and Group 2 by unpaired $t$ test.

*Plasma TSH, FT3, and FT4 concentrations*

The plasma TSH profile was depressed in hyperthyroid group than in control group. Plasma TSH levels remained unaltered during acute venous zinc administration in the hyperthyroid group (Figure 1B). In contrast, plasma TSH decreased significantly in the control group (Figure 1B), and there was a significant correlation between serum zinc and TSH after zinc administration, r= 0.9850, $p$<0.001 (Figure 1A and 1B; Table II). Venous zinc administration did not change the already elevated plasma levels of FT3 and FT4 in hyperthyroid group, as well as in control group (Figure 1C and 1D).

### Discussion

Alteration in thyroid function has been reported in zinc deficient status [13,14], and thyroid hormones have been shown to influence zinc metabolism at several levels, including absorption, transport, distribution, and secretion [7,9,15,16]. In this respect, zinc-deficient rats presented low serum TSH, T3, and T4 when compared to the ad libitum controls [2,3]. On the other hand, hypothyroidism induced zinc deficiency in humans [9,10], as well as zinc deficiency causes hypothyroidism [17]. The exact role of zinc in regulating thyroid hormone metabolism is not known. In our experiment, we administered intravenously a mean of 22.86 mg $Zn^{++}/m^2$ to healthy individuals and 21.70 mg $Zn^{++}/m^2$ to hyperthyroid patients (Table I) to observe the zinc effects on the plasma levels of TSH, FT3, and FT4. The low plasma levels of TSH and high plasma levels of FT3 and FT4 were expected in the hyperthyroid patients [1]. Because of that, the statistical analysis showed significant differences among TSH, FT3, and FT4 when comparing control individuals versus hyperthyroid patients, $p$<0.0001 (Figure 1B, 1C, and 1D; Table II).

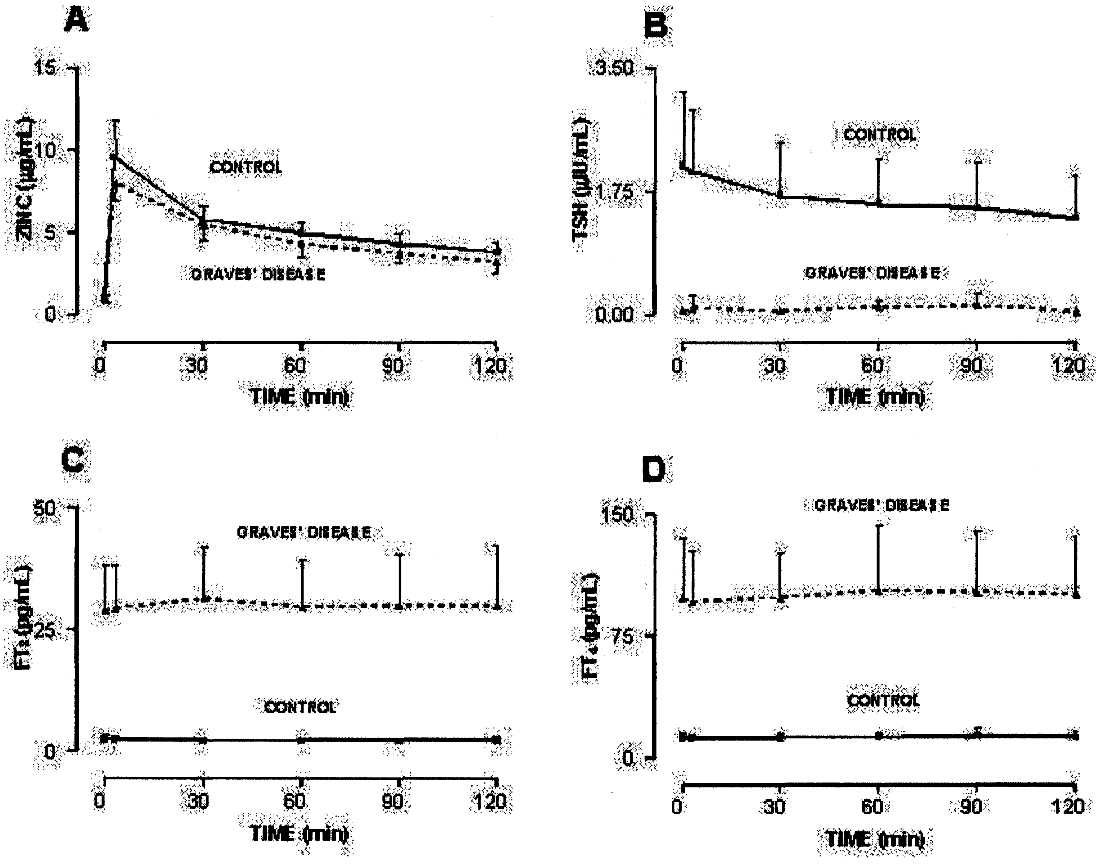

Figure 1. Levels of zinc, TSH, FT3 and FT4 in normal individuals and 12 patients with Graves' disease during venous zinc tolerance test (25 mg $Zn^{++}$). The results are represented as mean ± SD. (A) Values of serum zinc. These two curves are not significantly different ($p$>0.05). (B) Values of plasma TSH. The two curves showing TSH levels are significantly different ($p$ <0.0001). In hyperthyroid patients, TSH levels are virtually not detectable, whereas in control individuals, TSH levels decreased after zinc administration. (C) Values of plasma FT3. These two curves are significantly different ($p$ <0.0001). Acute zinc administration did not change the plasma FT3 profile in both control and hyperthyroid groups. (D) Values of plasma FT4. Both plasma FT4 profiles are significantly different ($p$ <0.0001), and again, not modified by acute zinc injection. All results are mean ± SD. (A): $p$ >0.05 vs. control by linear regression and unpaired $t$ test, and (B), (C) and (D): $p$ <0.0001 vs. control by unpaired $t$ test.

However, acute zinc administration did not modify the plasma TSH, FT3, and FT4 profiles in the hyperthyroid group, but decreased the plasma TSH profile in the control group (Figure 1B, 1C, and 1D). This phenomenon could not be attributed to the circadian rhythm, since correlation analysis revealed the existence of a strong positive correlation between zinc and TSH (r= 0.9850, $p$<0.001). The levels of FT3 and FT4 remained unalterable because the fall in plasma TSH probably cannot affect the free fractions of these hormones in 120 min [1]. Moreover, zinc did not significantly change the plasma TSH levels in hyperthyroid patients because of the extremely low levels of this hormone (0.08 ± 0.03 µIU/mL) during the test period. This is the first time that an experiment like that was performed in humans (healthy and hyperthyroid individuals) injecting pharmacological doses of zinc. In this regard, only zinc supplementation was realized in experimental animals. For example, this metal decreased hepatic 5'-deiodinase activity and significantly reduced serum T4 levels in obese and lean mice [11]. Some sites of regulation of thyroid hormones by zinc have been proposed, including peripheral deiodination of T4 [4,13], binding of T3 to nuclear receptors [5,18], synthesis of TRH [2,4], and cell membrane stabilization [19]. However, we only observed the acute zinc-induced decrement of plasma TSH levels in healthy individuals.

In conclusion, this study indicated that zinc administered acutely and in pharmacological doses does not alter the plasma levels of TSH, FT3, and FT4 in Graves' disease patients, but decreases TSH levels in healthy individuals.

**Acknowledgments**
This work was partially supported by FAPESP (# 95/5431-0 and 96/06733-2) and CNPq (# 520763-95-5 and 524130-96-5). We thank Cláudia L. H. Vasques, Elizabeth R. Calaço, and Pasqualina N.F. Moreira for technical assistance.

**References**

[1] P.R. Larsen, T.F. Davies, I.D. Hay, in "Williams Textbook of Endocrinology", W.B. Saunders Co., Philadelphia, (1998) pp. 389-515
[2] J.E. Morley, J. Gordon, J.M. Hershman, *Am. J. Clin. Nutr.* **33** (1980), 1767.
[3] A.E. Pekary, H.C. Lukaski, I. Mena, S.M. Smith, S. Bhasin, J.M. Hershman, *Peptides* **14** (1993), 315.
[4] A.E. Pekary, H.C. Lukaski, I. Mena, J.M. Hershman, *Peptides* **12** (1991), 1025.
[5] M.I. Surks, I.J. Ramirez, L.E. Shapiro, M. Kumara-Siri, *J. Biol. Chem.* **262** (1989), 9820.
[6] I. Nishi, R. Kawate, T. Usui, *Postgrad. Med. J.* **56** (1980), 833.
[7] K. Yoshida, Y. Kiso, T. Watanabe, K. Kaise, N. Kaise, Y. Itagaki, M. Yamamoto, T. Sakurada, K. Yoshinaga, *Metabolism* **39** (1990),182.
[8] S. Nishiyama, Y. Futagoishi-Suginohara, M. Matsukura, T. Nakamura, A. Higashi, M. Shinohara, I. Matsuda, *J. Am. Coll. Nutr.* **13** (1994), 62.
[9] W.P. Pimenta, J. Brandão-Neto, P.R. Curi, *Trace Elem. Med.* **9** (1992), 34.
[10] W.L. Dai, R.I. Henkin, *Clin Res* **42** (1994), A172.
[11] M.-D. Chen, P.-Y. Lin, W.-H. Lin, *Biol. Trace Elem. Res.* **61** (1998), 89.
[12] J. Brandão-Neto, G.M.F.S. Mazeto, A.V.B. Castro, T. Shuhama, N.B. Figueiredo, R.J.S. Franco, P.R. Curi, in "Metal Ions in Biology and Medicine", John Libbey Eurotext, Paris, (1996), pp. 663-666.
[13] J.W. Oliver, D.S. Sachan, P. Su, F.M. Applehans, *Drug-Nutr. Interact.* **5** (1987), 113.
[14] H.C. Lukaski, C.B. Hall, M.J. Marchello, *Horm. Metab. Res.* **24** (1992), 363.
[15] K. Aihara, Y. Nishi, S. Hatano, M. Kihara, K. Yoshimitsu, N. Takeichi, T. Ito, H. Ezaki, T. Usui, *Am. J. Clin. Nutr.* **40** (1984), 26.
[16] E. Dolev, P.A. Deuster, B. Solomon, U.H. Trostmann, L. Wartofsky, K.D. Burman, *Metabolism* **37** (1988), 61.
[17] F. Licastro, E. Mocchegiani, M. Zannotti, G. Arena, M. Masi, N. Fabris, Intern. *J. Neuroscience* **65** (1992), 259.
[18] I.J. Ramirez, M. Halmer, L.E. Shapiro, M. Surkes, *Horm. Metab. Res.* **23** (1991), 155.
[19] J. Brandão-Neto, G. Madureira, B.B. Mendonça, W. Bloise, A.V.B. Castro, *Biol. Trace Elem. Res.* **49** (1995), 139.

**Received: May 24, 2000 - Accepted: June 14, 2000 -**
**Received in publishable format: June 20 2000**

